# Mapping QTLs for early leaf spot resistance and yield component traits using an interspecific AB-QTL population in peanut

**DOI:** 10.3389/fpls.2024.1488166

**Published:** 2025-01-16

**Authors:** J. Gomis, A. Sambou, J. R. Nguepjop, H. A. Tossim, M. Seye, R. Djiboune, D. Sambakhe, D. Loko, S. Conde, M. H. Alyr, D. J. Bertioli, S. C. M. Leal-Bertioli, J. F. Rami, A. Kane, D. Fonceka

**Affiliations:** ^1^ Département de Biologie Végétale, Faculté des Sciences et Techniques, Université Cheikh Anta Diop de Dakar, Dakar, Senegal; ^2^ Institut Sénégalais de Recherches Agricoles (ISRA/Centre d’Etude Regional pour l’Amélioration de l’Adaptation à la Sécheresse (CERAAS), Thies, Senegal; ^3^ CIRAD, UMR AGAP, Montpellier, France; ^4^ CIRAD, INRAE, AGAP, University Montpellier, Institut Agro, Montpellier, France; ^5^ Institute of Plant Breeding, Genetics and Genomics, University of Georgia, Athens, GA, United States; ^6^ Department of Crop and Soil Sciences, University of Georgia, Athens, GA, United States; ^7^ Department of Plant Pathology, University of Georgia, Athens, GA, United States

**Keywords:** peanut, early leaf spot resistance, wild crop relative, *Arachis*, AB-QTL, marker assisted selection

## Abstract

Early leaf spot (ELS), caused by *Passalora personata* (syn. *Cercospora arachidicola*), is a highly damaging peanut disease worldwide. While there are limited sources of resistance in cultivated peanut cultivars, wild relatives carry alleles for strong resistance, making them a valuable strategic resource for peanut improvement. So far, only a few wild diploid species have been utilized to transfer resistant alleles to cultivars. To mitigate the risk of resistance breakdown by pathogens, it is important to diversify the sources of resistance when breeding for disease resistance. In this study, we created an AB-QTL population by crossing an induced allotetraploid (IpaCor1), which combines the genomes of the diploid species *Arachis ipaënsis* and *A. correntina*, with the susceptible cultivar Fleur11. *A. correntina* has been reported to possess strong resistance to leaf spot diseases. The AB-QTL population was genotyped with the Axiom-Arachis 48K SNPs and evaluated for ELS resistance under natural infestation over three years in Senegal. Marker/trait associations enabled the mapping of five QTLs for ELS resistance on chromosomes A02, A03, A08, B04, and B09. Except for the QTL on chromosome B09, the wild species contributed favorable alleles at all other QTLs. One genomic region on chromosome A02 contained several relevant QTLs, contributing to ELS resistance, earliness, and increased biomass yield, potentially allowing marker-assisted selection to introduce this region into elite cultivars. This study’s findings have aided in diversifying the sources of resistance to ELS disease and other important agronomic traits, providing another compelling example of the value of peanut wild species in improving cultivated peanut.

## Introduction

The peanut, also known as groundnut, originates from South America. It is grown in over a hundred countries, with a global production of about 54.2 million tons on 30.5 million hectares in 2022 (FAOSTAT, 2024). More than 80% of the peanut is produced in Asia and Africa, with China, India, and Nigeria being the top three peanut producers (FAOSTAT, 2024). Peanut is a valuable food, with high protein content (22%-30%), edible oil (44%-56%), vitamins (E, K, and B), and minerals (Ca, P, Mg, Zn, and Fe) (Nigam, 2014). In developing countries, groundnuts are primarily crushed to produce oil and serve as a vital cash crop for farmers. In developed countries, whole peanuts are used to produce peanut butter, candies, and salted snacks.

The cultivated peanut *Arachis hypogaea* (genome composition AABB) belongs to the genus Arachis, section Arachis, which includes 29 wild diploid species and the wild tetraploid form of peanut (*A. monticola*). These two tetraploid peanut species originated from a natural hybridization event between two wild diploid species, *A. duranensis* (AA) and *A. ipaënsis* (BB), accompanied by spontaneous chromosome doubling (2n = 4x = 40) (Kochert et al., 1996; Seijo et al., 2004; Favero et al., 2006; Burow et al., 2009; Bertioli et al., 2020).

Peanut production is limited by several biotic and abiotic factors, especially in developing countries where it is mainly grown under rainfall and low-input conditions. Among the major biotic constraints is Early Leaf Spot (ELS), a fungal disease caused by *Passalora personata* (syn. *Cercospora arachidicola*) (McDonald et al., 1985; Shokes and Culbreath, 1997). Significant yield losses have been attributed to this disease (Waliyar, 1990; Singh et al., 2011; Alidu et al., 2019). While elite cultivars generally have low to moderate levels of resistance to ELS, wild diploid species exhibit stronger resistance to various biotic stresses; they are valuable genetic resources for peanut improvement (Fávero et al., 2009; Leal-Bertioli et al., 2009, 2015; Upadhyaya et al., 2011; De Blas et al., 2021). Among the wild diploid species, *A. cardenasii* has been extensively used in breeding programs to develop cultivars resistant to multiple diseases, including ELS (Abdou et al., 1974; Stalker, 1984; P. Subrahmanyam et al., 1985; Leal-Bertioli et al., 2009; Bertioli et al., 2021a). In the 1970s, a foundational study crossed *A. cardenasii* (accession GKP 10017) with PI 261942-3, a cultivated variety from Bolivia. Several 40-chromosome interspecific hybrids were recovered and extensively tested for ELS resistance (Stalker, 1984). Of the thirteen hybrids tested, 10 demonstrated high resistance to ELS, and were used worldwide to enhance late leaf spot (LLS) resistance in elite cultivars. To date, *A. cardenasii* remains the most widely used wild source of resistance to leaf spot diseases (Bertioli et al., 2021a). Although there have been few reports of a breakdown in the leaf spot resistance conferred by *A. cardenasii* genetics, using new diploid species as sources of resistance would expand options for breeders and should help avoid resistance breakdown.

Resistance to ELS has been described in wild relatives of cultivated peanut, including *A. stenosperma*, *A. batizocoi*, *A. diogoi*, *A. villosa*, *A. magna*, *A. duranensis*, *A. valida*, *A. ipaënsis*, *A. monticola*, and *A. correntina* (Abdou et al., 1974; Subrahmanyam, 1985; Fávero et al., 2009; Gonzales et al., 2022). However, the use of wild species to enhance *A. hypogaea* has historically been limited by ploidy differences between wild and cultivated species and the presence of linkage drag. These obstacles have been overcome by the development of fertile-induced allotetraploids that can be crossed with cultivated peanut, along with the availability of thousands of molecular markers thanks to the peanut genome sequence (Bertioli et al., 2016, 2019). The number of synthetic allotetraploids released and used in breeding programs is increasing dramatically (Fonceka, 2010; Mallikarjuna et al., 2011; Leal-Bertioli et al., 2015; Nguepjop, 2016; Nguepjop et al., 2016; Sambou, 2017; Stalker, 2017; Ballén-Taborda et al., 2019; Bertioli et al., 2021b; Chu et al., 2021; Essandoh et al., 2022; Gonzales et al., 2022). Recently, a synthetic allotetraploid combining the genomes of the diploid species *A. ipaënsis* and *A. correntina* (IpaCor1) showed strong resistance to early and late leaf spots (Gonzales et al., 2022). This indicates that this synthetic could be a good source for moving alleles that confer ELS resistance from the wild to the cultivated species.

Tanksley et al. (1996) proposed an advanced backcross strategy, which allows for the simultaneous identification and transfer of favorable alleles from exotic germplasms to elite breeding lines. This strategy has had a positive impact on crop breeding programs. It has been successfully used in the breeding of economically important crops such as tomato (Stevens et al., 2007; Kwabena Osei et al., 2022), wheat (Kunert et al., 2007; Kaur et al., 2018), sorghum (Rao et al., 1999; Tao et al., 2018), rice (Bimpong et al., 2011; Jiang et al., 2020), cotton (Draye et al., 2005; Chandnani et al., 2018), barley (Li et al., 2006; Xu et al., 2018), and also peanut (Fonceka et al., 2012; Varshney et al., 2014; Ballén-Taborda et al., 2019; Kumari et al., 2020; Denwar et al., 2021; Essandoh et al., 2022). In addition, significant advances have been made in genomics-assisted breeding with the development of the Axiom_Arachis 48K SNP array (Korani et al., 2019), providing peanut breeding programs with a large number of useful molecular markers for rapid and efficient tracking of favorable alleles.

In this study, we crossed the synthetic tetraploid IpaCor1 (*A. ipaënsis* K 30076 x *A. correntina* GKP 9548)^4x^ with the susceptible cultivar Fleur11 to develop an inter-specific Advanced Backcrossed-QTL (AB-QTL) population. This material has been assessed for three consecutive years to evaluate its resistance to ELS and agronomic performance. We constructed a SNP-based genetic map, which, in combination with phenotypic data, was used to map QTLs for ELS resistance and yield-related traits. Wild species’ potential contributions to improving these traits are discussed.

## Material and methods

### Plant material

An advanced backcross QTL (AB-QTL) population of 220 individuals was developed by crossing the cultivated variety Fleur11 with IpaCor1 (*A. ipaensis* K30076 x *A. correntina* GKP9548)^4x^. Fleur11 was the recurrent female parent, while IpaCor1, developed at Georgia University (Gonzales et al., 2022), was the male parent. Fleur11 is a short-cycle peanut variety with an erect growth habit, drought tolerance, and high yield, but it is susceptible to leaf spot diseases such as ELS and LLS. It was introduced in Senegal from China in 1990. IpaCor1 resists early leaf spot disease (Gonzales et al., 2022). To develop the population, Fleur11 was first crossed with IpaCor1, and true F_1_ plants were identified using SSR molecular markers. These F_1_ were then backcrossed to Fleur11 to generate 116 BC_1_F_1_ plants, also identified with SSR molecular markers. Twenty BC_1_F_1_ were randomly selected and used as males to cross with Fleur11 to produce the BC_2_F_1_ generation. All BC_1_F_1_ were also self-pollinated to generate the BC_1_F_2_ generation. A first round of self-pollination was conducted on the BC_2_F_1_ and the BC_1_F_2_ plants to produce BC_2_F_2_ and the BC_1_F_3_ plants, which were individually genotyped before being self-pollinated again to yield 114 BC_2_F_3_ and 116 BC_1_F_4_ families used for phenotyping. The different stages of population development took place in controlled greenhouse conditions at the “Centre d’Etude Regional pour l’Amélioration de l’Adaptation à la Secheresse” (CERAAS) in Thies, Senegal. The plants were cultivated in large pots filled with sandy soil. The susceptible control used was the Chromosome Segment Substitution Line (CSSL) 12CS_048, derived from the cross Fleur11 x (*A. ipaensis* x *A. duranensis*)^4x^ (Fonceka, 2010; Fonceka et al., 2012). Additionally, CS16, an introgression line carrying QTLs for resistance to early and late leaf spot and rust on chromosomes A02 and A03 inherited from *A. cardenasii* (Stalker, 2017; Clevenger et al., 2018; Bertioli et al., 2021a), was used as the resistant control.

### Experimental design

The population was phenotyped during three consecutive rainy seasons between 2020 and 2022 at the ISRA research station located at Nioro du RIP, Kaolack, Senegal (13°45’ N, 15°47’ W). The experimental design used for each trial was an alpha lattice design. In 2020, only 153 lines were evaluated in three replications and nine blocks of 17 lines per block due to seed limitation. In 2021 and 2022, all 220 lines of the AB-QTL population were evaluated in three replications and fifteen blocks of 17 lines per block. Each line had ten seeds sown per row. The rows were three meters long with thirty centimeters of space between plants on the row and fifty centimeters between rows. The two checks (12CS_048 and CS16) were repeated in each block of all three replications, and the recurrent parent Fleur11 was repeated five times in each replication.

### Phenotypic characterization

Flowering dates, ELS resistance, and yield components like total biomass per plant, pod weight per plant, haulm weight per plant, hundred pod weight, hundred seed weight, pod length, and pod width were assessed.

#### Flowering dates

The number of days from sowing to the appearance of the first flower (FlwD1) and to the flowering of 50% of the plants (FlwD50%) were counted and recorded for each line.

#### Early leaf spot resistance

**Figure 1:**
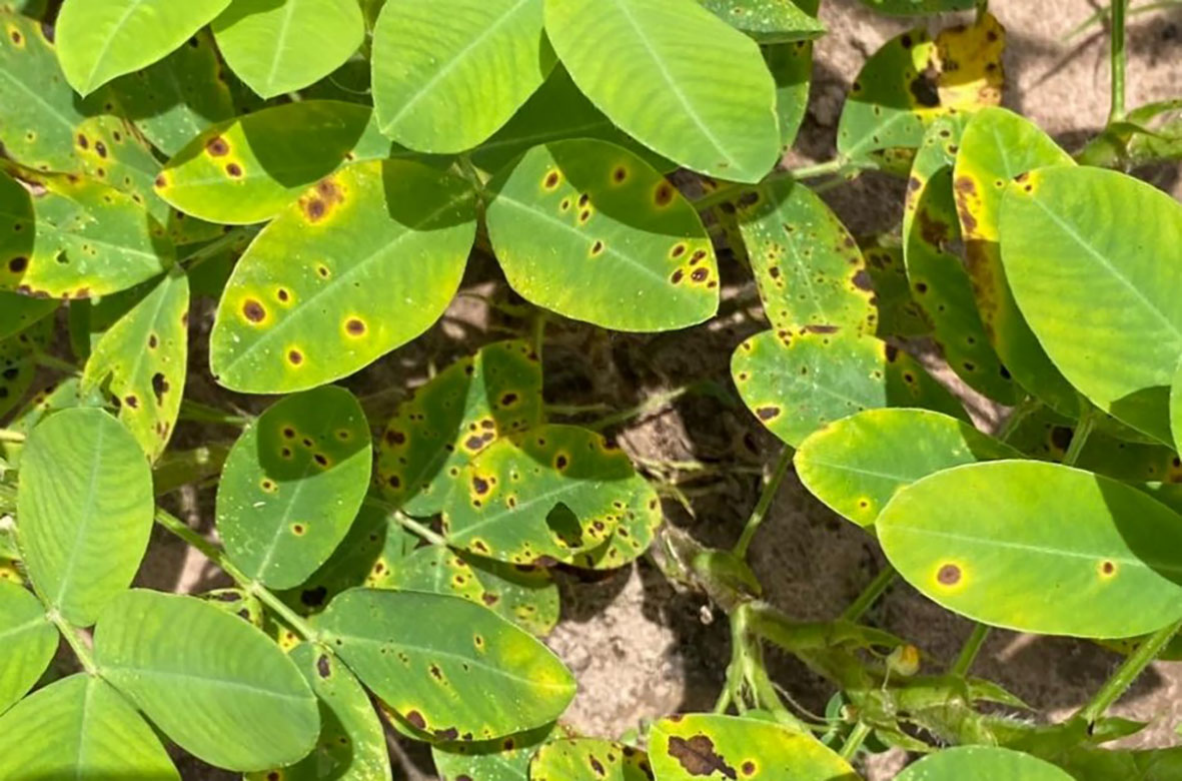
ELS symptoms on Fleur11 (60 days after sowing, Nioro du RIP, Senegal, 2022).


Early Leaf spot (ELS) severity was evaluated using the modified 0-9 scale method proposed by (Subrahmanyam, 1985). Scores were given according to the disease’s severity on plants. Data were collected every ten days, starting from the appearance of the first disease symptoms characterized by necrotic dark-brown lesions surrounded by a yellow halo on the upper side of the leaves (**Figure 1**). These scores were used to calculate the Area Under Diseases Progress Curves (AUDPC) according to the formula proposed in Simko and Piepho (2012):

$$\text{AUDPC}=\sum_{i=1}^{n-1}\left(\frac{y_{i}+y_{i+1}}{2}\times \left(t_{i+1}-t_{i}\right)\right)$$

where y_i_ and t_i_ are the ordinal disease’s score and the number of days at the i^th^ observation, respectively, and n is the total number of observations.

#### Yield components

Yield component traits were collected after drying the plants at ambient temperature for one month. The total biomass was first weighted to determine the total biomass per plant (TB). The pods were removed and weighted to determine the total pod weight per plant (PW). The haulm weight per plant (HW) was calculated as the difference between the total biomass and the total pod weight. One hundred pods were randomly sampled and weighted (HPW). These pods were shelled, and all seeds contained in mature pods characterized by a dark internal pericarp color were counted and weighted. The weight of 100 seeds (HSW) was calculated by multiplying the weight of all mature seeds by one hundred and dividing the result by the number of mature seeds. Pod width (PW) and pod length (PL) were characterized using image analysis. A sample of thirty pods per line was randomly selected and scanned on a flatbed scanner. The resulting images were analyzed using the Rigatoni (Rami, 2022) R package to determine the pods’ length and width. Pod width and pod length were only determined in the 2020 trial.

### Statistical analyses

Statistical analyses were performed using the software Breeding View (BV) and R. The normality of the residuals was tested and descriptive statistics (mean, minimum, maximum, standard deviation) were generated. The analysis of variance (ANOVA) was performed to estimate the effect of the genotypes and of the replications on each trait following a mixed model with the genotypes as fixed effects and the replications and blocs as random effects.


Yijk=μ+Gi+Rj+Bjk+eijk

with Yijk = observed value for a given trait, μ = mean of the population, Gi = genotype effect, Rj = replication effect, Bjk = block within replication effect and eijk = residual error.

The broad sense heritability for each trait and the BLUEs (Best Linear Unbiased Estimators) for each genotype were estimated from the model. BLUEs were extracted and used for QTL mapping.

### Genotyping

The AB-QTL population and the two parents Fleur11 and IpaCor were genotyped using the Affymetrix Axiom_Arachis 48K SNP array (Clevenger et al., 2018; Korani et al., 2019). DNA was extracted at CERAAS using the MATAB (Mixed Alkyl Trimethyl Ammonium Bromide) protocol as described by (Nguepjop, 2016) from 100 mg of dried leaves harvested on 21-day-old plants. Genotypic data were analyzed using the Axiom Analysis Suite 2.0 software. A set of polymorphic SNP markers that presented less than 10% distortion of segregation, as determined using a χ^2^ test, and less than 10% of missing data was selected.

### Genetic map construction and QTL detection

A genetic map was constructed using IciMapping software version 4.2. Polymorphic markers selected according to the listed criteria above were used for genetic map construction. The grouping and ordering of markers to construct linkage groups were determined using marker information, recombination frequency, and estimation of genetic distance. QTLs mapping was performed with R/qtl package version 1.66 (Broman et al., 2003) using the Haley-Knott regression method implemented in the “scanone” function. Because of the specific family structure of our advanced backcross population, particularly the BC_2_F_3_ lines that were derived from 20 BC_1_F_1_, a family covariate effect was added as co-factor in the QTL detection model. One thousand permutation tests were used and a QTL was declared when the LOD score was over the threshold value at the 5% level. For each of the QTL, the percentage of phenotypic variation explained and the effects were estimated using the “fitqtl” function. Detected QTLs were visualized on the genetic map using Spidermap v1.7.1 software (J.-F. Rami – 2017, unpublished).

## Results

### Traits variability and heritability

**Figure 2:**
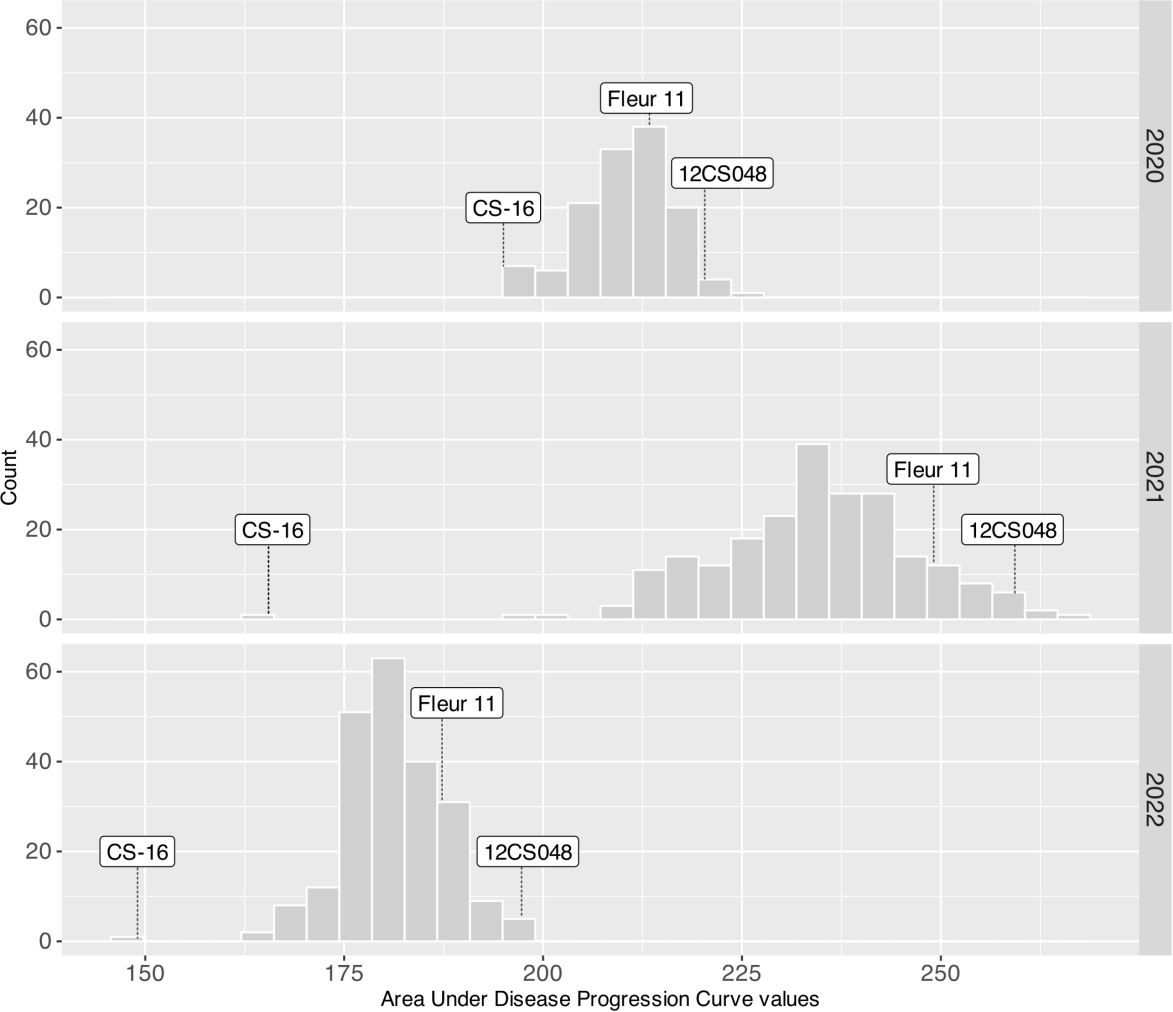
ELS severity variation in 2020, 2021 and 2022. Arrows indicate the phenotypic values of CS16 (resistant control), 12CS_048 (susceptible control) and Fleur11 (recurrent parent).

All traits exhibited significant variation (P<0.001) between genotypes. The broad sense heritability was estimated for each trait every year and ranged from 0.52 to 0.98 (**Table 1**). Phenotypic data were normally distributed for all traits, including early leaf spot severity (**Figure 2**).

**Table 1:** Summary of trait’s variation and heritability.

Traits	Name	Years	Min.	Max.	Mean	Variance	Pvalue	H^2^
		2020	14	28	22	4.63	<0,001	0.88
**FlwD1**	Date to first flower appearance	2021	15	25	20	3.4	<0,001	0.87
		2022	18	26	22	3	<0,001	0.84
		2020	18	31	24	5.11	<0,001	0.98
**FlwD50%**	Date to 50% flowering	2021	17	28	23	3.7	<0,001	0.87
		2022	17	30	24	3	<0,001	0,82
		2020	180	245	210	155	<0,001	0.62
**AUDPC**	Area under disease progress curve	2021	155	300	232	983	<0,001	0.68
		2022	150	220	180	303	<0,001	0.58
		2020	25.1	259.1	126.1	2122	<0,001	0.7
**TBP**	Total plant biomass	2021	31.9	209.7	115.2	1227.1	<0,001	0.52
		2022	22	109	65	257	<0,001	0.78
		2020	3.44	110.7	36.74	350.8	<0,001	0.74
**PWP**	Plant pod weight	2021	3.4	44.3	20.6	75.1	<0,001	0.65
		2022	0.8	23.1	9.6	22	<0,001	0.81
		2020	12.17	178.5	87.99	1158	<0,001	0.67
**HWP**	Plant haulm weight	2021	23.5	166.7	92.2	832.2	<0,001	0.53
		2022	20	92	55	200	<0,001	0.79
		2020	18.6	155.5	85.44	639.9	<0,001	0.93
**HPW**	100-pod weight	2021	35.3	139.3	87.3	367.7	<0,001	0.66
		2022	34	113	73	239	<0,001	0.78
		2020	16.85	57.26	36.9	77.45	<0,001	0.93
**HSW**	100-seed weight	2021	18.9	49.2	34.1	29.4	<0,001	0.67
		2022	23	48	35	24	<0,001	0.76
**PL**	Pod length	2020	21.45	35.18	28.15	6.79	<0,001	0.92
**PW**	Pod width	2020	7.49	13.15	10.37	1.49	<0,001	0.95

### Genotyping and genetic map construction

**Figure 3:**
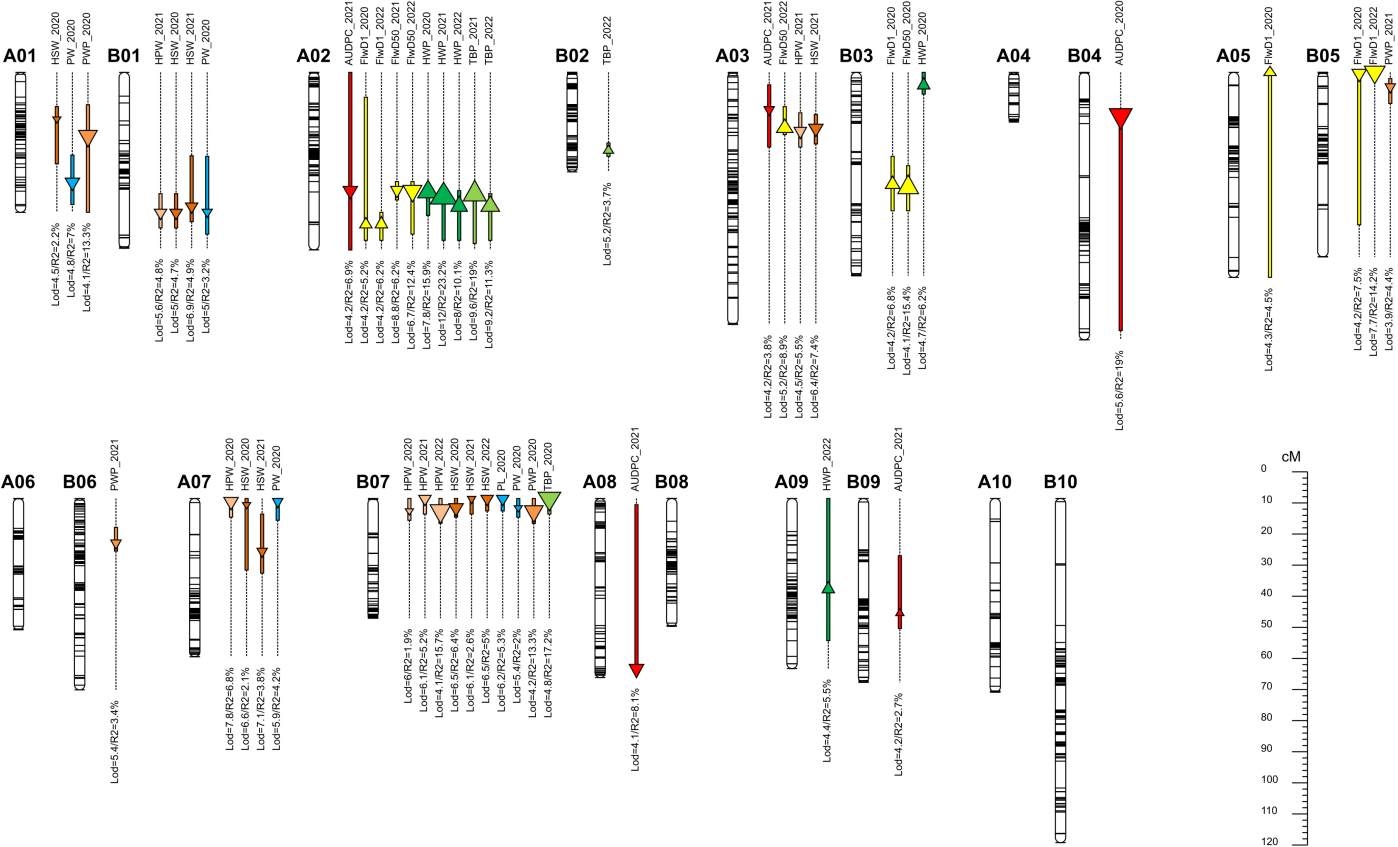
Graphical representation of identified QTLs on the genetic map. Each QTL is represented by a box spanning the QTL confidence interval and a triangle located at the LOD peak with an area proportional to the R^2^. Upward triangles represent a positive effect from IpaCor1 and downward triangles a positive effect from Fleur11.

Out of the 48,000 SNPs on the Axiom_Arachis 48K SNP array, 95% were removed due to a lack of marker polymorphisms, a high percentage of missing data, and/or segregation distortion patterns. Finally, we used 2329 highly reliable SNP markers to create the genetic map. These markers were placed into 20 linkage groups, covering a total genetic distance of 1142.83 cM. On the A sub-genome, 1135 markers were assigned, covering a genetic distance of 532.67 cM, with the number of markers per chromosome varying from 28 for A04 to 191 for A02. On the B sub-genome, 1194 SNPs were mapped, covering a genetic distance of 610.16 cM, with the number of SNPs per chromosome ranging from 68 for B05 to 190 for B10. The genetic map with identified QTLs is shown in **Figure 3**.

### QTLs identification

Forty-eight (48) QTLs were detected for ten (10) traits across the three years. At least one QTL was identified per trait. Some QTL regions were common to different traits while several QTLs for the same trait were identified in homeologous and non-homeologous regions. The name of the QTLs was formed by combining the abbreviation of the trait name, the chromosome name, and the position of the QTL on the chromosome. A summary of detected QTLs with their effects is shown in **Table 2**.

**Table 2:** Summary of the identified QTLs and their effects.

QTLs	Trait	Year	thr	chr	pos	marker	ci.low	ci.high	lod	R^2^	add	dom
**qAUDPC.B04_14.0**	Area under disease progress curve	2020	3.97	B04	14.0	AX-176814723	12.00	83.00	5.57	18.96	-1.79	8.76
**qAUDPC.A02_38.0**		2021	3.86	A02	38.0	AX-147215161	0.00	57.06	4.17	6.88	-3.70	-3.42
**qAUDPC.A03_12.0**		2021	3.86	A03	12.0	AX-176821377	4.00	24.00	4.18	3.77	-2.84	1.55
**qAUDPC.A08_54.9**		2021	3.86	A08	54.9	AX-176802655	2.00	56.55	4.08	8.06	-3.83	0.01
**qAUDPC.B09_37.0**		2021	3.86	B09	37.0	AX-177637406	18.44	41.81	4.19	2.66	2.03	7.68
**qFlwD1.A02_48.9**	Days to first flower appearance	2020	3.98	A02	48.9	AX-147215183	8.00	54.00	4.24	5.18	0.52	-0.27
**qFlwD1.A05_0.0**		2020	3.98	A05	0.0	AX-176814782	0.00	65.93	4.33	4.50	0.44	-1.01
**qFlwD1.B05_0.4**		2020	3.98	B05	0.4	AX-176819116	0.00	49.00	4.17	7.51	-0.76	-0.54
**qFlwD1.B03_36.0**		2020	3.98	B03	36.0	AX-176823645	27.00	44.48	4.20	6.82	0.72	-0.55
**qFlwD1.A02_48.9**		2022	4.13	A02	48.9	AX-147215183	45.00	54.00	4.23	6.18	0.64	-0.27
**qFlwD1.B05_0.0**		2022	4.13	B05	0.0	AX-176823282	0.00	1.29	7.70	14.20	-1.03	-0.26
**qFlwD50.B03_37.0**	Days to 50% fowering	2020	4.11	B03	37.0	AX-176823645	30.00	44.48	4.14	15.37	1.35	-1.49
**qFlwD50.A02_38.0**		2021	4.17	A02	38.0	AX-147215161	35.09	41.00	8.80	6.21	-0.13	1.30
**qFlwD50.A02_38.0**		2022	4.29	A02	38.0	AX-147215161	35.09	52.00	6.74	12.37	-0.42	1.27
**qFlwD50.A03_18.0**		2022	4.29	A03	18.0	AX-176806296	11.00	20.00	5.21	8.88	0.42	-0.52
**qHPW.A07_1.0**	100-Pod weight	2020	4.02	A07	1.0	AX-147226922	0.00	6.00	7.83	6.85	-4.59	-15.21
**qHPW.B07_4.0**		2020	4.02	B07	4.0	AX-176791817	0.00	7.00	5.96	1.93	-6.59	-11.86
**qHPW.A03_19.0**		2021	3.82	A03	19.0	AX-147215661	13.00	24.00	4.54	5.49	-2.77	0.66
**qHPW.B07_0.0**		2021	3.82	B07	0.0	AX-177639234	0.00	5.00	6.15	5.22	-3.91	-5.46
**qHPW.B01_45.0**		2021	3.82	B01	45.0	AX-176795252	39.00	50.00	5.55	4.81	-4.25	2.32
**qHPW.B07_4.0**		2022	4.02	B07	4.0	AX-176791817	0.00	8.00	4.07	15.67	-2.61	-10.91
**qHSW.A07_2.0**	100-Seed weight	2020	3.96	A07	2.0	AX-147226922	0.00	23.00	6.56	2.14	-0.97	-2.86
**qHSW.B07_3.0**		2020	3.96	B07	3.0	AX-176791817	0.00	6.00	6.52	6.39	-2.48	-8.31
**qHSW.A01_15.0**		2020	3.96	A01	15.0	AX-147209800	11.00	29.41	4.47	2.15	-1.32	4.10
**qHSW.B01_45.0**		2020	3.96	B01	45.0	AX-176795252	39.00	50.00	5.01	4.69	-3.08	-2.38
**qHSW.A03_18.0**		2021	3.97	A03	18.0	AX-176806296	13.46	23.00	6.40	7.37	-0.90	-0.05
**qHSW.A07_17.1**		2021	3.97	A07	17.1	AX-176804662	5.00	24.00	7.07	3.79	-1.00	0.23
**qHSW.B07_0.0**		2021	3.97	B07	0.0	AX-177639234	0.00	5.00	6.14	2.58	-0.78	-0.81
**qHSW.B01_43.0**		2021	3.97	B01	43.0	AX-176821996	26.83	48.00	6.89	4.89	-1.11	-0.17
**qHSW.B07_0.0**		2022	4.04	B07	0.0	AX-177639234	0.00	4.00	6.51	5.00	-0.81	-2.72
**qHWP.A02_38.5**	Plant haulm weight	2020	4.52	A02	38.5	AX-147215161	37.00	46.00	7.79	15.95	2.76	20.83
**qHWP.B03_4.0**		2020	4.52	B03	4.0	AX-176803111	0.00	7.00	4.72	6.15	7.53	6.47
**qHWP.A02_40.0**		2021	4.04	A02	40.0	AX-147215161	37.00	54.00	11.95	23.18	4.84	8.26
**qHWP.A02_43.0**		2022	3.99	A02	43.0	AX-147215161	38.00	54.00	7.96	10.13	5.56	0.20
**qHWP.A09_29.1**		2022	3.99	A09	29.1	AX-176803649	0.00	45.74	4.39	5.47	2.87	1.43
**qPL.B07_0.0**	Pod length	2020	3.90	B07	0.0	AX-177639234	0.00	4.00	6.20	5.28	-1.22	-0.04
**qPW.A07_1.0**	Pod width	2020	4.09	A07	1.0	AX-147226922	0.00	7.00	5.88	4.23	-0.21	-0.61
**qPW.B07_3.0**		2020	4.09	B07	3.0	AX-176791817	0.00	6.00	5.37	1.98	-0.34	-0.41
**qPW.A01_35.4**		2020	4.09	A01	35.4	AX-176813023	26.56	42.48	4.85	7.02	-0.36	0.56
**qPW.B01_45.0**		2020	4.09	B01	45.0	AX-176795252	27.00	52.00	4.98	3.24	-0.34	-0.35
**qPWP.B07_4.0**	Plant pod weight	2020	4.08	B07	4.0	AX-176791817	0.00	8.00	4.23	13.25	-6.50	0.63
**qPWP.A01_20.4**		2020	4.08	A01	20.4	AX-176809386	11.0	45.0	4.14	13.52	-4.24	2.63
**qPWP.B06_14.4**		2021	3.89	B06	14.4	AX-176805464	9.3	16.9	5.35	5.27	-0.88	-1.88
**qPWP.B10_79.3**		2021	3.89	B10	79.3	AX-177637104	73.0	81.0	4.77	1.48	-0.55	0.58
**qTBP.B07_0.0**	Plant total biomass	2020	3.84	B07	0.0	AX-177639234	0.0	4.0	4.98	17.65	-14.90	-0.47
**qTBP.A02_39.0**		2021	4.05	A02	39.0	AX-147215161	37.0	56.0	9.56	14.64	5.26	5.85
**qTBP.A02_43.0**		2022	3.93	A02	43.0	AX-147215161	39.0	54.0	9.20	11.28	6.82	-0.53
**qTBP.B02_25.1**		2022	3.93	B02	25.1	AX-147241813	22.7	27.0	5.15	3.75	1.41	8.46

thr: LOD threshold for declaring QTLs at 5%, chr: chromosome name, pos: QTL peak in cM, ci.low and ci.high: confidence intervals, lod: QTL lod, R^2^: phenotypic variation explained, add: additivity, dom: dominance.

### Early leaf spot resistance QTLs

Five (5) QTLs were detected for early leaf spot resistance on linkage groups A02, A03, A08, B04, and B09 explaining 3% to 19% of the phenotypic variation. Among the five QTLs, one was detected in 2020 (qAUDPC.B04_14.0) and the other four (qAUDPC.A02_38.0, qAUDPC.A03_12.0, qAUDPC.A08_54.9, qAUDPC.B09_37.0) were identified in 2021. No QTL was mapped in 2022. Except for the QTL on chromosome B09, the wild alleles were associated with early leaf spot resistance on all other chromosomes. The AB-QTL population’s structure allowed for the inclusion and estimation of a dominance effect in the QTL detection model, as it displayed an average of 6% heterozygous genotypes at a given marker. Four out of the five QTLs detected for AUDPC showed a significant dominance effect (**Supplementary Figure S1**). In most cases, dominance was in favor of the sensitivity allele, except for the QTL on A02, where the heterozygous class exhibited the same level of resistance as the wild homozygous class.

### Flowering dates QTLs

Ten (10) QTLs were detected for flowering time traits across the three years of experimentation. For the days to the appearance of the first flower (FlwD1), 6 QTLs were mapped on chromosomes A02, A05, B03, and B05 explaining 5% to 14% of the phenotypic variation. The QTLs qFlwD1.A02_48.9 and qFlwD1.B05_0.4 were detected in 2020 and 2022 while qFlwD1.A05_0.0 was only detected in 2020. For the QTLs detected on A02 and A05 the wild alleles extended the number of days to the appearance of the first flower, while on B05 it was associated with early flowering. For the date to fifty percent flowering, 4 QTLs were detected on A02, A03, and B03 chromosomes explaining 6% to 15% of the phenotypic variation. The QTL, qFlwD50%.A02_38.5 was detected in 2021 and 2022 while qFlwD50%.B02_31.4 and qFlwD50%.A03_18.0 were respectively identified in 2021 and 2022. For QTLs on A02 and B02, the wild alleles were associated with a decrease in the number of days to fifty percent flowering. Conversely, qFlwD50%.A03_18.0 was associated with the increase in the number of days to fifty percent flowering.

### Yield components QTLs

A total of thirty-three (33) QTLs were identified for the yield-related traits across the three years. For total biomass per plant, four (4) QTLs were detected on chromosomes A02, B02, and B07 explaining 4% to 18% of the phenotypic variation. The two QTLs qTBP.A02_39.0 and qTBP.A02_43.0 on chromosome A02 were detected in 2021 and 2022, respectively, while qTBP.B07_0.0 was identified in 2020 and qTBP.B02_25.1 was identified in 2022. The wild alleles contributed to the increase of the total biomass per plant on chromosomes A02 and B02 and a decrease on chromosome B07. For haulm weight, five (5) QTLs were identified on A02, A09 and B03 and the phenotypic variation explained ranged from 5% to 23%. The QTLs qHWP.A02_38.5 and qHWP.B03_4.0 were detected in 2020, qHWP.A02_40.0 in 2021 and the remaining two qHWP.A02_43.0 and qHWP.A09_29.1 were identified in 2022. At all QTLs, the wild alleles were associated with an increase in the haulm weight. For pod weight per plant four (4) QTLs were detected on chromosomes A01, B06, B07, and B10 explaining 1.5% to 14% of the phenotypic variation. The two QTLs qPWP.A01_20.4 and qPWP.B07_4.0 were mapped in 2020, while those on chromosomes B06 and B10, qPWP.B06_14.4 and qPWP.B10_79.3, were detected only in 2021. The wild alleles contributed negatively at all the QTLs by decreasing the pod weight. Six (6) QTLs were identified for hundred pod weight on chromosomes A03, A07, B01, and B07 explaining 2% to 16% of the phenotypic variation. The QTL qHPW.B07_4.0 was consistently detected in 2020 and 2022 in addition to qHPW.A07_1.0 also detected in 2020. Three QTLs were mapped in 2021 on chromosomes A03, B01, and B07, qHPW.A03_19.0, qHPW.B01_45.0, and qHPW.B07_0.0 respectively. The wild alleles were associated with a decrease of the hundred pod weight at all the QTLs. Nine (9) QTLs were identified for the hundred seed weight trait on chromosomes A01, A03, A07, B01, and B07 explaining 2% to 7% of the observed variation. On B07, three QTLs were detected, qHSW.B07_3.0 (2020) and qHSW.B07_0.0 (2021 and 2022). On A07, qHSW.A07_2.0 was detected in 2020, and qHSW.A07_17.1 in 2021. On homeologous chromosomes A01 and B01 two QTLs were detected in 2020 qHSW.A01_15.0 and HSW.B01_45.0 while in 2021, qHSW.A03_18.0 and qHPW.B01_43.0 were identified. For all identified QTLs, the wild alleles were associated with a decrease in the hundred seed weight value. One (1) QTL was identified for pod length (PL) on chromosome B07 (qPL.B07_0.0) explaining 5% of the phenotypic variation. The wild allele had a negative effect by reducing pod length. Four (4) QTLs were identified for pod width (PW) on homeologous chromosomes A01 and B01 (qPW.A01_35.4 and qPW.B01_45.0), A07 and B07 (qPW.A07_1.0 and qPW.B07_3.0) explaining 2% to 7% of the phenotypic variation. At all QTLs, the wild allele had negative effects by reducing the value of the pod width.

## Discussion

Crop wild relatives are valuable sources of genetic variation and new alleles to improve crop production. Their beneficial contributions have been documented across crops like wheat, millet, rice, maize, barley, tomato, chickpea, potato, and peanut (Huang et al., 2003; Rao et al., 2003; Hajjar and Hodgkin, 2007; Leal-Bertioli et al., 2009; Fonceka, 2010; Bertioli et al., 2011; Nguepjop, 2016; Sambou, 2017; Stalker, 2017; Tossim et al., 2020). In this study, we have developed an interspecific AB-QTL population by crossing a synthetic tetraploid, which combines the wild diploid genomes of *A. ipaënsis* and *A. correntina*, and the cultivar Fleur11. Over three years of phenotypic characterization, several lines outperformed Fleur11 in ELS resistance, earliness, and haulm weight, highlighting the positive contribution of wild species. For pod weight per plant (PWP), hundred pod weight (HPW), and hundred seed weight (HSW), the top-performing genotypes were comparable to Fleur11.

### Wild alleles contributed positive variation for ELS resistance, early flowering, and to a lesser extent yield component traits

Peanut wild relatives often exhibit high resistance to diverse pathogens. Several studies using interspecific populations have reported QTLs for late leaf spot (Khedikar et al., 2010; Sujay et al., 2012; Nguepjop, 2016; Shirasawa et al., 2018; Essandoh et al., 2022), rust (Khedikar et al., 2010; Kolekar et al., 2016; Shirasawa et al., 2018), and ELS (Stalker and Mozingo, 2001; Khera et al., 2016; Han et al., 2018). These studies emphasize the potential of peanut wild species for improving the cultivated varieties. In our study five QTLs for ELS were mapped, three on the A sub-genome (qLS.A02_38.0, qLS.A03_12.0, and qLS.A08_54.9) and two on the B sub-genome (qLS.B04_14.0 and qLS.B09_37.0). Wild alleles contributed to disease resistance for all QTLs except for one on chromosome B09. Notably, none of the ELS resistance QTLs were consistently detected in all years, mainly because of the variations in disease pressure from year to year. This variability limited the effectiveness of QTL detection, especially under low disease pressure. For instance, the disease pressure was high in 2021 with AUDPC (Area Under Disease Progress Curve) values ranging from 155 to 300, low in 2020, and very low in 2022 with AUDPC values of 180-245 and 150-220, respectively (**Figure 2**). As a result, four out of the five QTLs were detected under high disease pressure in 2021, while one was identified under low disease pressure in 2020. Moreover, in 2021, the QTL mapped on chromosome B04 had a major effect, since it explained more than 10% of the phenotypic variation.

In this study, we found that *Arachis correntina* (GKP9548) showed positive variation in ELS resistance, with three QTLs mapped on chromosomes A02, A03, and A08. The QTL region on chromosome A02 is similar to the one found in CS16 (the resistant check we used in this study and that is a derivative of *Arachis cardenasii* (GKP 10017) introgression line). While CS16 is primarily known for its resistance to late leaf spots (Khedikar et al., 2010; Kolekar et al., 2016; Stalker, 2017; Agarwal et al., 2018; Bertioli et al., 2021a; Lamon et al., 2021), it has also been used in the past to develop resistant lines to early leaf spot disease (Stalker, 1984). Our study also revealed that none of the AB-QTL lines performed better than CS16 in terms of early leaf spot resistance, even if a few were almost as resistant as CS16 under the high disease-pressure environment. These results indicate that *A. correntina* (GKP9548) could serve as a new source of resistance to ELS, potentially reducing the risk of resistance breakdown and improving cultivated varieties.

Improving agronomic traits such as early maturity, high yield, and yield components is crucial in rising to the challenge of increasing world population and changing climate conditions. In our study, we have identified forty-three QTLs associated with flowering dates (FlwD1 and FlwD50%) and yield components (TBP, PWP, HWP, HPW, HSW, PL, and PW). Twenty-eight percent of these QTLs carried beneficial alleles from wild species, underscoring the value of wild species in enhancing the agronomic performance of cultivated varieties. Similar results were described in tomatoes where twenty percent of the detected QTLs for 19 agronomic traits had favorable alleles coming from the wild parent *Lycopersicon hirsutum* (Bernacchi et al., 1998). In wheat, wild alleles have been used to improve yield by around twenty-five percent under saline conditions (Kaur et al., 2018). A QTL analysis in peanut using an interspecific population revealed ninety-five QTLs for agronomic traits, with nearly half having beneficial alleles from the wild species *A. duranensis* and *A. ipaënsis* (Fonceka et al., 2012). In our study, most of the identified QTLs for agronomic traits had minor effects, explaining less than 10% of the phenotypic variation. This result may be due to the polygenic nature of yield-related traits, often controlled by multiple QTLs with small effects (Wang et al., 2018). It may also be explained by the simultaneous presence of both positive and negative effect QTLs, with the latter masking the effects of the former, particularly in less backcrossed interspecific populations. For the QTLs linked to above-ground biomass yield (TBP and HWP), the wild alleles had positive effects, while for those related to pod and seed’s yield and size (i.e. PWP, HPW, HSW, PL, and PW), the wild species contributed negatively. This suggests that different genomic regions control above-ground biomass yield and pod and seed yield and size. Therefore, selecting cultivated parent alleles for pod and seed-related QTLs and wild parent alleles for above-ground biomass yield would be beneficial for developing dual-purpose cultivars, which are particularly important for farmers in the Sahelian countries of West Africa.

### Co-location of QTLs

The co-location of QTLs governing different traits has been described by several authors (Fonceka et al., 2012; Nguepjop, 2016; Luo et al., 2017; Wang et al., 2018; Khera et al., 2019). In our study, we found a compelling example of co-location on chromosome A02 where QTLs for FlwD50%, ELS disease, TBP, and HWP overlapped. Notably, in this region, wild alleles from *A. correntina* contributed to early flowering, ELS resistance, and increased biomass weight. This could be explained by several genes underlying this QTL region, each controlling these traits, or that one gene with pleiotropic effects is at play (Wang et al., 2018; Wu et al., 2019). We also observed consistent QTLs across years in this region, except for ELS. Additionally, the QTLs for FlwD50%, ELS disease, TBP, and HWP were all linked to the same molecular marker “AX-147215161”. Selecting this region using closely linked markers around that SNP may lead to the development of early maturing, resistant, and productive lines. Another interesting co-location of QTLs involves the pod and seed-related QTLs on chromosomes A07 and B07, as previously reported (Fonceka et al., 2012; Luo et al., 2018; Chavarro et al., 2020). In these regions, the wild alleles were responsible for reducing the pod and seed weight and size. Similar results were reported in a study by Fonceka et al. (2012), where 10 to 26% of the pod and seed size reduction was attributed to the wild allele on chromosome A07. These findings were further confirmed by Tossim et al. (2020). Fine mapping of this wild genomic region on chromosome A07 identified putative genes that may be involved in peanut domestication (Alyr et al., 2020).

Another example of QTL co-location is found on chromosome A03, where QTLs for ELS resistance, FlwD50%, HPW, and HSW were identified. While the wild alleles contributed to disease resistance, they also reduced HPW and HSW, and caused late flowering, indicating a linkage drag in this region.

## Conclusion

In this study, we used an AB-QTL population created by crossing the synthetic tetraploid IpaCor1 with the cultivated variety Fleur11 to map QTLs for ELS (Early Leaf Spot) resistance and agronomic traits. We identified five QTLs linked to ELS resistance on chromosomes A02, A03, A08, B04, and B09. Resistance was associated with wild alleles at all QTL regions except on chromosome B09. *A. correntina* emerged as a major source of ELS resistance that can be utilized to improve cultivated peanut. Additionally, twenty-eight percent of the detected QTLs for agronomic traits had favorable alleles coming from the wild parent. Notably, on chromosome A02, the ELS QTL co-localized with several other QTLs for flowering precocity, haulm, and pod production, making this region a strong candidate for marker-assisted improvement of elite peanut cultivars.

## Data Availability

The original contributions presented in the study are publicly available. This data can be found here: https://dataverse.cirad.fr/dataset.xhtml?persistentId=doi:10.18167/DVN1/8LYUWO.

## Supplementary material

Supplementary Table S1 Details of the genetic map.

Supplementary Figure S1 Graphical representation of dominance effect of detected QTLs for AUDPC. AA and BB represent homozygous genotypes for cultivated and wild parents respectively and AB is for heterozygous genotypes.

## References

[B1] Abdou Y. A.-M. Gregory W. C. Cooper W. E. (1974). Sources and nature of resistance to *cercospora arachidicola* hori and *cercosporidium personatum* (Beck & Curtis) deighton in *arachis* species 1. Peanut Sci. 1, 6–11. doi: 10.3146/i0095-3679-1-1-3

[B2] Agarwal G. Clevenger J. Pandey M. K. Wang H. Shasidhar Y. Chu Y.. (2018). High-density genetic map using whole-genome resequencing for fine mapping and candidate gene discovery for disease resistance in peanut. Plant Biotechnol. J. 16, 1954–1967. doi: 10.1111/pbi.12930 29637729 PMC6181220

[B3] Alidu M. S. Abukari S. Abudulai M. (2019). Screening groundnut (*Arachis hypogaea*) genotypes for resistance to early and late leaf spot diseases. J. Exp. Agric. Int. 37, 1–9. doi: 10.9734/jeai/2019/v37i430274

[B4] Alyr M. H. Pallu J. Sambou A. Nguepjop J. R. Seye M. Tossim H. A.. (2020). Fine-mapping of a wild genomic region involved in pod and seed size reduction on chromosome a07 in peanut (*Arachis hypogaea* L.). Genes 11, 1–23. doi: 10.3390/genes11121402 PMC7761091 33255801

[B5] Ballén-Taborda C. Chu Y. Ozias-Akins P. Timper P. Holbrook C. C. Jackson S. A.. (2019). A new source of root-knot nematode resistance from *Arachis stenosperma* incorporated into allotetraploid peanut (*Arachis hypogaea*). Sci. Rep. 9, 1–13. doi: 10.1038/s41598-019-54183-1 31776412 PMC6881346

[B6] Bernacchi D. Beck-Bunn T. Eshed Y. Lopez J. Petiard V. Uhlig J.. (1998). Advanced backcross QTL analysis in tomato. I. Identification of QTLs for traits of agronomic importance from *Lycopersicon hirsutum* . Theor. Appl. Genet. 97, 381–397. doi: 10.1007/s001220050908

[B7] Bertioli D. J. Abernathy B. Seijo G. Clevenger J. Cannon S. B. (2020). Evaluating two different models of peanut’s origin. Nat. Genet. 52, 557–559. doi: 10.1038/s41588-020-0626-1 32393860

[B8] Bertioli D. J. Cannon S. B. Froenicke L. Huang G. Farmer A. D. Cannon E. K. S.. (2016). The genome sequences of *Arachis duranensis* and *Arachis ipaensis*, the diploid ancestors of cultivated peanut. Nat. Genet. 48, 438–446. doi: 10.1038/ng.3517 26901068

[B9] Bertioli D. J. Clevenger J. Godoy I. J. Stalker H. T. Wood S. Santos J. F.. (2021a). Legacy genetics of *Arachis cardenasii* in the peanut crop shows the profound benefits of international seed exchange. Proc. Natl. Acad. Sci. 118, e2104899118. doi: 10.1073/pnas.2104899118 34518223 PMC8463892

[B10] Bertioli D. J. Gao D. Ballen-Taborda C. Chu Y. Ozias-Akins P. Jackson S. A.. (2021b). Registration of GA-BatSten1 and GA-MagSten1, two induced allotetraploids derived from peanut wild relatives with superior resistance to leaf spots, rust, and root-knot nematode. J. Plant Regist. 15, 372–378. doi: 10.1002/plr2.20133

[B11] Bertioli D. J. Jenkins J. Clevenger J. Dudchenko O. Gao D. Seijo G.. (2019). The genome sequence of segmental allotetraploid peanut *Arachis hypogaea* . Nat. Genet. 51, 877–884. doi: 10.1038/s41588-019-0405-z 31043755

[B12] Bertioli D. J. Seijo G. Freitas F. O. Valls J. F. M. Leal-Bertioli S. C. M. Moretzsohn M. C. (2011). An overview of peanut and its wild relatives. Plant Genet. Resour. 9, 134–149. doi: 10.1017/S1479262110000444

[B13] Bimpong I. K. Serraj R. Chin J. H. Ramos J. Mendoza E. M. Hernandez J. E.. (2011). Identification of QTLs for drought-related traits in alien introgression lines derived from crosses of rice (*Oryza sativa* cv. IR64) × *O. glaberrima* under lowland moisture stress. J. Plant Biol. 54, 237–250. doi: 10.1007/s12374-011-9161-z

[B14] Broman K. W. Wu H. Sen Ś Churchill G. A. (2003). R/qtl: QTL mapping in experimental crosses. Bioinformatics. 19, 889–890. doi: 10.1093/bioinformatics/btg112 12724300

[B15] Burow M. D. Simpson C. E. Faries M. W. Starr J. L. Paterson A. H. (2009). Molecular biogeographic study of recently described B- and A-genome *Arachis* species, also providing new insights into the origins of cultivated peanut. Genome 52, 107–119. doi: 10.1139/g08-094 19234559

[B16] Chavarro C. Chu Y. Holbrook C. Isleib T. Bertioli D. Hovav R.. (2020). Pod and seed trait QTL identification to assist breeding for peanut market preferences. G3: Genes Genomes Genet. 10, 2297–2315. doi: 10.1534/g3.120.401147 PMC7341151 32398236

[B17] Chu Y. Stalker H. T. Marasigan K. Levinson C. M. Gao D. Bertioli D. J.. (2021). Registration of three peanut allotetraploid interspecific hybrids resistant to late leaf spot disease and tomato spotted wilt. J. Plant Regist. 15, 562–572. doi: 10.1002/plr2.20146

[B18] Clevenger J. Chu Y. Chavarro C. Botton S. Culbreath A. Isleib T. G.. (2018). Mapping late leaf spot resistance in peanut (*Arachis hypogaea*) using QTL-seq reveals markers for marker-assisted selection. Front. Plant Sci. 9. doi: 10.3389/fpls.2018.00083 PMC5807350 29459876

[B19] Draye X. Chee P. Jiang C.-X. Decanini L. Delmonte T. A. Bredhauer R.. (2005). Molecular dissection of interspecific variation between *Gossypium hirsutum* and *G. barbadense* (cotton) by a backcross-self approach: II. Fiber fineness. Theor. Appl. Genet. 111, 764–771. doi: 10.1007/s00122-005-2061-1 15995865

[B20] Essandoh D. A. Odong T. Okello D. K. Fonceka D. Nguepjop J. Sambou A.. (2022). Quantitative Trait Analysis Shows the Potential for Alleles from the Wild Species *Arachis batizocoi* and *A. duranensis* to Improve Groundnut Disease Resistance and Yield in East Africa. Agron 12, 2202. doi: 10.3390/agronomy12092202

[B21] FAOSTAT (2024). Available online at: https://www.fao.org/faostat/fr/data/QCL (Accessed February 28, 2024).

[B22] Fávero A. P. Moraes S. A. Garcia A. A. F. Valls J. F. M. Vello N. A. (2009). Characterization of rust, early and late leaf spot resistance in wild and cultivated peanut germplasm. Sci. Agric. (Piracicaba Braz.) 66, 110–117. doi: 10.1590/S0103-90162009000100015

[B23] Favero A. P. Simpson C. E. Valls J. F. M. Vello N. A. (2006). Study of the Evolution of Cultivated Peanut through Crossability Studies among *Arachis ipaensis*, *A. duranensis*, and *A. hypogaea* . Crop Sci. 46, 1546–1552. doi: 10.2135/cropsci2005.09-0331

[B24] Fonceka D. (2010). Elargissement de la base génétique de l’arachide cultivée (*Arachis hypogaea*): Applications pour la construction de populations, l’identification de QTL et l’amélioration de l’espèce cultivée (Montpellier, France: Montpellier SupAgro). Available online at: https://www.theses.fr/2010NSAM0023 (Accessed February 28, 2024).

[B25] Fonceka D. Tossim H.-A. Rivallan R. Vignes H. Faye I. Ndoye O.. (2012). Fostered and left behind alleles in peanut: interspecific QTL mapping reveals footprints of domestication and useful natural variation for breeding. BMC Plant Biol. 12, 26. doi: 10.1186/1471-2229-12-26 22340522 PMC3312858

[B26] Gonzales M. Kemerait R. Bertioli D. Bertioli S. (2022). Strong resistance to early and late leaf spot in peanut-compatible wild-derived induced allotetraploids. Plant Dis. doi: 10.1094/PDIS-03-22-0721-RE 35748737

[B27] Hajjar R. Hodgkin T. (2007). The use of wild relatives in crop improvement: a survey of developments over the last 20 years. Euphytica 156, 1–13. doi: 10.1007/s10681-007-9363-0

[B28] Han S. Yuan M. Clevenger J. P. Li C. Hagan A. Zhang X.. (2018). A SNP-based linkage map revealed QTLs for resistance to early and late leaf spot diseases in peanut (*Arachis hypogaea* L.). Front. Plant Sci. 9. doi: 10.3389/fpls.2018.01012 PMC6048419 30042783

[B29] Huang X. Q. Cöster H. Ganal M. W. Röder M. S. (2003). Advanced backcross QTL analysis for the identification of quantitative trait loci alleles from wild relatives of wheat (*Triticum aestivum* L.). Theor. Appl. Genet. 106, 1379–1389. doi: 10.1007/s00122-002-1179-7 12750781

[B30] Kaur S. Jindal S. Kaur M. Chhuneja P. (2018). “Utilization of Wild Species for Wheat Improvement Using Genomic Approaches,” in Biotechnologies of Crop Improvement, Volume 3: Genomic Approaches. Eds. Gosal S. S. Wani S. H. (Springer International Publishing, Cham), 105–150. doi: 10.1007/978-3-319-94746-4_6

[B31] Khedikar Y. P. Gowda M. V. C. Sarvamangala C. Patgar K. V. Upadhyaya H. D. Varshney R. K. (2010). A QTL study on late leaf spot and rust revealed one major QTL for molecular breeding for rust resistance in groundnut (*Arachis hypogaea* L.). Theor. Appl. Genet. 121, 971–984. doi: 10.1007/s00122-010-1366-x 20526757 PMC2921499

[B32] Khera P. Pandey M. K. Mallikarjuna N. Sriswathi M. Roorkiwal M. Janila P.. (2019). Genetic imprints of domestication for disease resistance, oil quality, and yield component traits in groundnut (*Arachis hypogaea* L.). Mol. Genet. Genomics 294, 365–378. doi: 10.1007/s00438-018-1511-9 30467595

[B33] Khera P. Pandey M. K. Wang H. Feng S. Qiao L. Culbreath A. K.. (2016). Mapping quantitative trait loci of resistance to tomato spotted wilt virus and leaf spots in a recombinant inbred line population of peanut (*Arachis hypogaea* L.) from sunOleic 97R and NC94022. PloS One 11, e0158452. doi: 10.1371/journal.pone.0158452 27427980 PMC4948827

[B34] Kochert G. Stalker H. T. Gimenes M. A. Galgaro M. L. Lopes C. R. Moore K. (1996). RFLP and cytogenetic evidence on the origin and evolution of allotetraploid domesticated peanut, *Arachis hypogaea* (Leguminosae). Am. J. Bot. 83, 1282–1291. doi: 10.1002/j.1537-2197.1996.tb13912.x

[B35] Kolekar R. M. Sujay V. Shirasawa K. Sukruth M. Khedikar Y. P. Gowda M. V. C.. (2016). QTL mapping for late leaf spot and rust resistance using an improved genetic map and extensive phenotypic data on a recombinant inbred line population in peanut (*Arachis hypogaea* L.). Euphytica 209, 147–156. doi: 10.1007/s10681-016-1651-0

[B36] Korani W. Clevenger J. P. Chu Y. Ozias-Akins P. (2019). Machine learning as an effective method for identifying true single nucleotide polymorphisms in polyploid plants. Plant Genome 12, 180023. doi: 10.3835/plantgenome2018.05.0023 PMC12962348 30951095

[B37] Kunert A. Naz A. A. Dedeck O. Pillen K. Léon J. (2007). AB-QTL analysis in winter wheat: I. Synthetic hexaploid wheat (*T. turgidum ssp. dicoccoides* × *T. tauschii*) as a source of favourable alleles for milling and baking quality traits. Theor. Appl. Genet. 115, 683–695. doi: 10.1007/s00122-007-0600-7 17634917

[B38] Lamon S. Chu Y. Guimaraes L. A. Bertioli D. J. Leal-Bertioli S. C. Santos J. F.. (2021). Characterization of peanut lines with interspecific introgressions conferring late leaf spot resistance. Crop Sci. 61, 1724–1738. doi: 10.1002/csc2.20414

[B39] Leal-Bertioli S. C. M. Cavalcante U. Gouvea E. G. Ballén-Taborda C. Shirasawa K. Guimarães P. M.. (2015). Identification of QTLs for rust resistance in the peanut wild species arachis magna and the development of KASP markers for marker-assisted selection. G3 5, 1403–1413. doi: 10.1534/g3.115.018796 25943521 PMC4502374

[B40] Leal-Bertioli S. Jose A. C. Alves-Freitas D. Moretzsohn M. Guimaraes P. Nielen S.. (2009). Identification of candidate genome regions controlling disease resistance in Arachis. BMC Plant Biol. 9, 112. doi: 10.1186/1471-2229-9-112 19698131 PMC2739205

[B41] Li J. Z. Huang X. Q. Heinrichs F. Ganal M. W. Röder M. S. (2006). Analysis of QTLs for yield components, agronomic traits, and disease resistance in an advanced backcross population of spring barley. Genome 49, 454–466. doi: 10.1139/g05-128 16767170

[B42] Luo H. Guo J. Ren X. Chen W. Huang L. Zhou X.. (2018). Chromosomes A07 and A05 associated with stable and major QTLs for pod weight and size in cultivated peanut (*Arachis hypogaea* L.). Theor. Appl. Genet. 131, 267–282. doi: 10.1007/s00122-017-3000-7 29058050

[B43] Luo H. Ren X. Li Z. Xu Z. Li X. Huang L.. (2017). Co-localization of major quantitative trait loci for pod size and weight to a 3.7 cM interval on chromosome A05 in cultivated peanut (*Arachis hypogaea* L.). BMC Genomics 18, 58. doi: 10.1186/s12864-016-3456-x 28068921 PMC5223410

[B44] Mallikarjuna N. Jadhav D. R. Reddy K. Husain F. Das K. (2011). Screening new Arachis amphidiploids, and autotetraploids for resistance to late leaf spot by detached leaf technique. Eur. J. Plant Pathol. 132, 17–21. doi: 10.1007/s10658-011-9859-2

[B45] McDonald D. Subrahmanyam P. Gibbons R. W. Smith D. H. (1985). Early and Late leafspot of groundnut. Information bulletin n°21. Patancheru, A.P (India: ICRISAT). Available online at: https://oar.icrisat.org/821/1/RA_00080.pdf (Accessed November 10, 2022).

[B46] Nguepjop R. J. (2016). Contribution des espèces sauvages à l’analyse génétique et à l’amélioration variétale de l’arachide cultivée (*Arachis hypogaea* L.) (Université Cheikh Anta Diop de Dakar). Available online at: http://bibnum.ucad.sn/viewer.php?c=ths&d=ths_2019_0044 (Accessed October 15, 2022).

[B47] Nguepjop J. R. Tossim H.-A. Bell J. M. Rami J.-F. Sharma S. Courtois B.. (2016). Evidence of genomic exchanges between homeologous chromosomes in a cross of peanut with newly synthetized allotetraploid hybrids. Front. Plant Sci. 7. doi: 10.3389/fpls.2016.01635 PMC5088615 27847512

[B48] Nigam S. N. (2014). Groundnut at a glance (Patancheru: ICRISAT). Available online at: http://oar.icrisat.org/8455/ (Accessed November 10, 2022).

[B49] Rami J.-F. (2022). Rigatoni. Available online at: https://github.com/jframi/rigatoni.

[B50] Rao D. G. Khanna-Chopra R. Sinha S. K. (1999). Comparative performance of sorghum hybrids and their parents under extreme water stress. J. Agric. Sci. 133, 53–59. doi: 10.1017/S0021859699006589

[B51] Rao N. K. Reddy L. J. Bramel P. J. (2003). Potential of wild species for genetic enhancement of some semi-arid food crops. Genet. Resour. Crop Evol. 50, 707–721. doi: 10.1023/A:1025055018954

[B52] Sambou A. (2017). Genetic Studies and Mapping of Beneficial Alleles from Wild Species to Improve Agronomic Traits in Cultivated Groundnut (*Arachis hypogaea* L.) - Sécheresse info. Available online at: http://www.secheresse.info/spip.php?article96371 (Accessed November 10, 2022).

[B53] Seijo J. G. Lavia G. I. Fernandez A. Krapovickas A. Ducasse D. Moscone E. A. (2004). Physical mapping of the 5S and 18S-25S rRNA genes by FISH as evidence that *Arachis duranensis* and *A. ipaensis* are the wild diploid progenitors of *A. hypogaea* (*Leguminosae*). Am. J. Bot. 91, 1294–1303. doi: 10.3732/ajb.91.9.1294 21652361

[B54] Shirasawa K. Bhat R. S. Khedikar Y. P. Sujay V. Kolekar R. M. Yeri S. B.. (2018). Sequencing analysis of genetic loci for resistance for late leaf spot and rust in peanut (*Arachis hypogaea* L.). Front. Plant Sci. 9. doi: 10.3389/fpls.2018.01727 PMC6275244 30534132

[B55] Shokes F. M. Culbreath A. K. (1997). “Early and Late Leaf Spots,” in Compendium of Peanut Diseases, 2nd Edition. Eds. Kokalis-Burelle N. Porter D. M. Rodriguez-Kabana R. Smith D. H. Subrahmanyam P. (APS Press, St. Paul, MN), 17–20.

[B56] Simko I. Piepho H. P. (2012). The area under the disease progress stairs: Calculation, advantage, and application. Phytopathology 102, 381–389. doi: 10.1094/PHYTO-07-11-0216 22122266

[B57] Singh M. P. Erickson J. E. Boote K. J. Tillman B. L. Jones J. W. Van Bruggen A. H. (2011). Late leaf spot effects on growth, photosynthesis, and yield in peanut cultivars of differing resistance. J. Agron. 103, 85–91. doi: 10.2134/agronj2010.0322

[B58] Stalker (1984). Utilizing *Arachis cardenasii* as a source of Cercospora leafspot resistance for peanut improvement. Euphytica 33, 529–538. doi: 10.1007/BF00021154

[B59] Stalker H. T. (2017). Utilizing wild species for peanut improvement. Crop Sci. 57, 1102–1120. doi: 10.2135/cropsci2016.09.0824

[B60] Stalker H. T. Mozingo L. G. (2001). Molecular markers of Arachis and marker-assisted selection. Peanut Sci. 28, 117–123. doi: 10.3146/i0095-3679-28-2-13

[B61] Stevens R. Buret M. Duffé P. Garchery C. Baldet P. Rothan C.. (2007). Candidate genes and quantitative trait loci affecting fruit ascorbic acid content in three tomato populations. Plant Physiol. 143, 1943–1953. doi: 10.1104/pp.106.091413 17277090 PMC1851805

[B62] Subrahmanyam P. (1985). Resistance to leaf spot caused by *cercosporidium personatum* in wild arachis species. Plant Dis. 69, 951. doi: 10.1094/pd-69-951

[B63] Subrahmanyam P. Ghanekar A. M. Nolt B. L. Reddy D. V. R. McDonald D. (1985). Resistance to Groundnut Diseases in Wild Arachis Species. Available online at: https://oar.icrisat.org/4161/1/CP_208.pdf (Accessed November 10, 2024).

[B64] Sujay V. Gowda M. V. C. Pandey M. K. Bhat R. S. Khedikar Y. P. Nadaf H. L.. (2012). Quantitative trait locus analysis and construction of consensus genetic map for foliar disease resistance based on two recombinant inbred line populations in cultivated groundnut (*Arachis hypogaea* L.). Mol. Breed. 30, 773–788. doi: 10.1007/s11032-011-9661-z 22924018 PMC3410029

[B65] Tanksley S. D. Grandillo S. Fulton T. M. Zamir D. Eshed Y. Petiard V.. (1996). Advanced backcross QTL analysis in a cross between an elite processing line of tomato and its wild relative L. pimpinellifolium. Theor. Appl. Genet. 92, 213–224. doi: 10.1007/BF00223378 24166170

[B66] Tao Y. Mace E. George-Jaeggli B. Hunt C. Cruickshank A. Henzell R.. (2018). Novel grain weight loci revealed in a cross between cultivated and wild sorghum. Plant Genome 11, 170089. doi: 10.3835/plantgenome2017.10.0089 PMC12810130 30025022

[B67] Tossim H.-A. Nguepjop J. R. Diatta C. Sambou A. Seye M. Sane D.. (2020). Assessment of 16 peanut (*Arachis hypogaea* L.) CSSLs derived from an interspecific cross for yield and yield component traits: QTL validation. Agron. 10, 583. doi: 10.3390/agronomy10040583

[B68] Upadhyaya H. D. Dwivedi S. L. Nadaf H. L. Singh S. (2011). Phenotypic diversity and identification of wild Arachis accessions with useful agronomic and nutritional traits. Euphytica 182, 103–115. doi: 10.1007/s10681-011-0518-7

[B69] Waliyar F. (1990). “Evaluation of yield losses due to groundnut leaf diseases in West Africa,” in *Summary Proceedings of the second ICRISAT regional groundnut meeting for West Africa*, Niamey, Niger (ICRISAT, Patancheru, India), 32–33.

[B70] Wang Z. Huai D. Zhang Z. Cheng K. Kang Y. Wan L.. (2018). Development of a high-density genetic map based on specific length amplified fragment sequencing and its application in quantitative trait loci analysis for yield-related traits in cultivated peanut. Front. Plant Sci. 9. doi: 10.3389/fpls.2018.00827 PMC6028809 29997635

[B71] Wu J. Chen P. Zhao Q. Cai G. Hu Y. Xiang Y.. (2019). Co-location of QTL for Sclerotinia stem rot resistance and flowering time in *Brassica napus* . Crop J. 7, 227–237. doi: 10.1016/j.cj.2018.12.007

