# Supplementary figures and images for: Mapping QTLs for early leaf spot resistance and yield component traits using an interspecific AB-QTL population in peanut
Source: Front Plant Sci. 2025 Jan 16;15:1488166. doi: 10.3389/fpls.2024.1488166 (PMC11779571; doi:10.3389/fpls.2024.1488166)

**A02@38**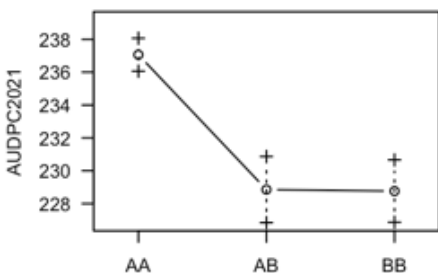

AX-147215161

**A03@12**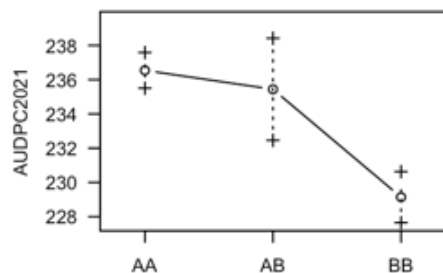

AX-176821377

**A08@54.9**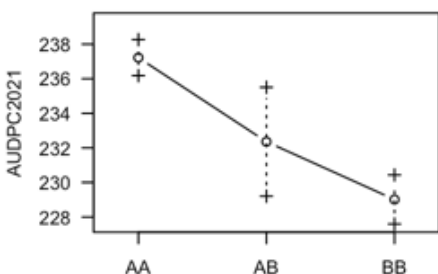

AX-176802655

**B09@37**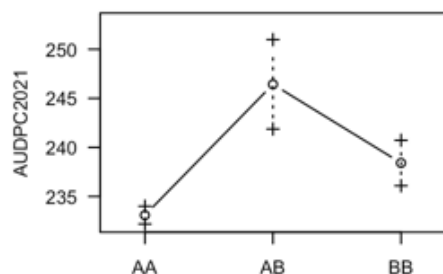

AX-177637406

**B04@14**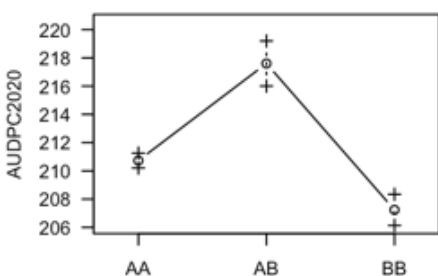

AX-176814723

Supplement: Supplementary Figure S1 — Graphical representation of dominance effect of detected QTLs for AUDPC. AA and BB represent homozygous genotypes for cultivated and wild parents respectively and AB is for heterozygous genotypes. [file DataSheet1.pdf]
